# Daily physical activity, human development index and insomnia in a representative sample of Brazilian adolescents: a cross-sectional analysis

**DOI:** 10.1590/1516-3180.2020.0745.R1.0604221

**Published:** 2021-08-09

**Authors:** Antônio Evaldo dos Santos, Raphael Henrique de Oliveira Araujo, Josiene Oliveira Couto, Danilo Rodrigues Pereira da Silva, Roberto Jerônimo dos Santos Silva

**Affiliations:** I MSc. Physical Education Professional, Department of Physical Education, Universidade Federal de Sergipe (UFS), São Cristóvão (SE), Brazil.; II MSc. Physical Education Professional, Department of Physical Education, Universidade Federal de Sergipe (UFS), São Cristóvão (SE), Brazil.; III MSc. Physical Education Professional, Department of Physical Education, Universidade Federal de Sergipe (UFS), São Cristóvão (SE), Brazil.; IV PhD. Physical Education Professional, Department of Physical Education, Universidade Federal de Sergipe (UFS), São Cristóvão (SE), Brazil.; V PhD. Physical Education Professional, Department of Physical Education, Universidade Federal de Sergipe (UFS), São Cristóvão (SE), Brazil.

**Keywords:** Exercise, Sleep initiation and maintenance disorders, Sleep, Adolescent, Mental health, Physical activity, Insomnia, Sleep habits, Youth

## Abstract

**BACKGROUND:**

Although there is a growing body of research pointing towards the need to investigate how different movement behaviors, such as physical activity and sleep, influence each other, the joint relationship between these factors and insomnia has been little explored among adolescents in developing countries.

**OBJECTIVES:**

To investigate the association between daily physical activity and insomnia in a national sample of Brazilian adolescents, according to the Human Development Index (HDI) of each Brazilian region.

**DESIGN AND SETTINGS:**

Cross-sectional study on 102,072 Brazilian students aged 11 to 19 years, selected from all regions of the country.

**METHODS:**

Information on insomnia and physical activity was self-reported by adolescents.

**RESULTS:**

Our analyses revealed that girls who accumulated at least 60 minutes/day of physical activity on up to three days/week were less prone to present insomnia. This pattern of association was maintained only for those who lived in high HDI regions (odds ratio, 0.87; 95% confidence interval, 0.75-0.99). For boys, there was a positive association between the number of active days and protection against insomnia, especially for those who lived in high HDI regions.

**CONCLUSION:**

Even amounts of physical activity that were lower than the weekly guidelines, were associated with better sleep quality for Brazilian adolescents, especially girls, and even for those who lived in regions with greater social and economic vulnerability.

## INTRODUCTION

Insomnia is defined as persistent difficulty in falling asleep and staying asleep, despite adequate sleep opportunities,^1^ and is considered to be the most common sleep disorder during adolescence, with an estimated prevalence ranging from 6% to 30% in this age group.^1^ Previous research has shown that insomnia is associated with symptoms of depression, substance abuse, poor school performance, mood disorders,^2^^,^^3^ social withdrawal and loneliness,^4^ lower emotional regulation,^5^^,^^6^ anxiety and suicidal thoughts.^7^ Despite the recommendations that adolescents should sleep for 8 to 10 hours per day,^8^ few young people achieve sufficient amounts of sleep,^9^ which converges on a secular trend of reduced sleep duration among children and adolescents.^10^ This scenario highlights insomnia as an important public health issue, as well as demonstrating the need to establish effective and suitable ways to address this problem during adolescence.

In this regard, the scientific literature provides arguments for different strategies for treating insomnia, such as cognitive-behavioral therapy and pharmacotherapy.^11^^,^^12^ However, although similar efficacy has been demonstrated between these therapies for treating insomnia,^13^ both of them are difficult to apply on a large scale, especially in the context of developing countries.^14^ Thus, studies have pointed towards physical activity (PA) as a possible low-cost strategy for reducing the effects of insomnia among adolescents.^15^ In a meta-analysis on 12 studies that investigated the relationship between PA and sleep among adolescents, it was concluded that individuals with higher PA levels, assessed subjectively and objectively, were more likely to present better sleep quality.^16^ Furthermore, the results from a clinical review demonstrated that moderate aerobic exercise training could be prescribed as pertinent non-pharmacological treatment for sleep disorders.^17^ Likewise, cross-sectional data from a survey of 14,782 American adolescents showed that only those who were active for at least 60 minutes/day, every day, presented an association with sufficient amounts of sleep (≥8 hours of sleep per night on an average school night).^18^ However, that analysis was not stratified by sex, which may have limited the interpretation of the results, since boys and girls differ in their PA levels and sleep quality.^19^

In addition, although research has shown that higher total PA levels are associated with better sleep quality,^16^^,^^18^^,^^20^ this relationship remains unclear. Studies carried out among adolescents in 33 European countries^21^ and in Canada^22^ did not find any association between higher PA levels and sleep disorders. Complementing these findings, the results from a longitudinal study on adolescents indicated that changes to accumulation of physical activity were unrelated to changes in mental health outcomes, such as mental distress,^23^ although the latter can be a confounder between physical activity and sleep disorders. However, considering the social and economic differences between developed and developing countries, there is a lack of information on how this association occurs among Brazilian adolescents, considering that the prevalence of PA varies according to a country’s level of development.^24^

Furthermore, a growing body of research has also pointed towards the need to investigate how different movement behaviors over a 24-hour period, such as PA, sedentary behavior (SB) and sleep, influence each other.^25^ Likewise, there is evidence that points towards the existence of a relationship between health-related behaviors, such as PA, SB and sleep, and urban and socioeconomic development.^26^ Previous research has shown that the features of both the social environment (family, social cohesion, safety, noise and socioeconomic status) and the physical environment (light exposure, traffic, air pollution and walkability) may influence sleep.^27^^,^^28^ However, the joint relationship between these factors and insomnia has not been explored.

## OBJECTIVES

The objectives of the present study were: 1) to investigate the association between daily PA and insomnia, in a national sample of Brazilian adolescents; and 2) to verify whether this association varies according to sex and the HDI of the region of this country.

## METHODS

### Study design and sample

This was a cross-sectional analysis based on data from the National School-based Health Survey (PeNSE in Portuguese), conducted in Brazil between April and September 2015. This survey had the aim of assessing the risk and protective factors relating to the health of students enrolled in public and private schools throughout Brazil.^29^ The sampling process and methods are detailed elsewhere.^29^ Briefly, PeNSE 2015 was the third edition of this survey, with previous editions conducted in 2009 and 2012. Unlike the other editions, the PeNSE 2015 database was composed of two different samples, named: sample 1, which included students in the 9^th^ grade of elementary school; and sample 2, composed of students aged 13 to 17 years, from the 6^th^ grade of elementary school up to the 3^rd^ grade of high school, in the reference year of this study.^29^ This analysis focuses on sample 1, due to the greater representativeness of these data on the students’ health.

For sample 1, students were selected through a complex cluster sampling process, which included schools in the 26 state capitals, plus the Federal District and 26 other municipalities, resulting in a total of 53 strata. In the capitals, the sampling process was carried out in two stages (schools as primary units and classes as secondary units), while in the other municipalities, stratification involved three stages (municipalities as primary units, schools as secondary units and classes as tertiary units). The sample size was calculated for each stratum, considering a sampling error of 3%, prevalence of 50% and confidence interval of 95%.^29^

Data were collected through a self-administered electronic questionnaire, composed of two main sections. The first section related to the school’s characteristics and was filled out by the principal or coordinator. The second section was self-administered by students and assessed their individual characteristics. Based on the 2013 school census, 3160 schools were selected and invited to participate in the study, totaling 4159 classes. All students in the classes selected were invited. In total, 3040 public and private schools, with 124,227 students, were considered eligible to participate in the study. Among the students present on the day of data collection, 102,301 (82%) agreed to participate in the study.^29^ Because of missing data, the current analysis considered data from 102,072 students (51.7% girls) aged from 11 to 19 years (mean age of 14.33 ± 1.06 years).

Informed consent was obtained from parents/legal guardians, and student assent was also obtained, as required by local ethics review boards. All procedures of PeNSE 2015 were approved by the National Research Ethics Committee of the National Health Council, and the study was conducted in accordance with the principles expressed in the Declaration of Helsinki (CONEP no. 1.006.467; dated March 30, 2015).

### Insomnia

The prevalence of insomnia was obtained through self-reports, by means of the following question: “*In the past 12 months, how often have you been unable to sleep at night because something worried you a lot?*” The response options were “*Never*”, “*Rarely*”, “*Sometimes*”, “*Most of the time*” or “*Always*”. The first three options were categorized as “No” = “0”. Individuals who answered “Most of the time” or “Always” were classified as having insomnia and were categorized as “Yes” = “1”. This categorization procedure was also used in a previous study.^20^

### Physical activity

Daily physical activity was self-reported through a validated questionnaire,^30^ using the following question: *“In the past seven days, on how many days did you exercise for at least 60 minutes a day?”* Daily physical activity was categorized according to the number of days reported (zero to seven).

### Geographical regions

The analyses were stratified according to the Human Development Index (HDI) of Brazil’s five macroregions. Thus, the Brazilian regions were classified as having low HDI (North = 0.667; Northeast = 0.663) = “0”; or as having high HDI (Southeast = 0.766; Center-West = 0.757; South = 0.754) = “1”.^31^ This form of classification is used in studies on regional inequalities in Brazil.^32^

### Covariables

The following covariables were considered: a) age group (< 14 years or ≥ 14 years); b) ethnicity, which in the present study was dichotomized as “white” or “non-white” (black, Asian, brown/mixed or indigenous); c) bullying, obtained through the question: “*Have you ever been bullied?*”, with the answer options “yes” or “no”. Those who answered “yes” were considered to be victims of bullying and were categorized as “1”; and d) television viewing, which was self-reported through the item: “*On an ordinary weekday, how many hours per day do you watch TV? (do not consider Saturday, Sunday or holidays)*”. The response options ranged from “I don’t watch TV” to “More than 8 hours per day” and the answers were categorized as “≤ 2 hours”, “> 2 to ≤4 hours” or “> 4 hours”.

### Statistical analysis

Descriptive statistics were presented as absolute and relative frequencies, stratified according to sex, along with confidence intervals (95% CI). Crude and adjusted binary logistic regression models, stratified according to sex and HDI of the Brazilian regions, were constructed in order to estimate the odds ratio (OR) and 95% CI. Only covariates with a P-value < 0.2 in the crude analyses were included in the final model. The statistical analyses were performed using the Statistical Package for the Social Sciences (SPSS) software, version 22.0 (IBM, Armonk, New York, United States), with statistical significance taken to be P ≤ 0.05.

## RESULTS

The descriptive characteristics of the sample according to sex are shown in Table 1. A total of 102,072 students (51.7% girls) participated in the study, with a mean age of 14.33 ± 1.06 years (ranging from 11 to 19 years), and most of them were “non-white” (> 65%). Almost 60% of the adolescents were from regions with low HDI. A greater proportion of the girls reported that they had been victims of bullying. Approximately one third of the adolescents reported watching TV for more than four hours per day. The prevalence of insomnia was more than twice as high for the girls (15.6%), compared with the boys. The proportion of the boys (13.4%) who declared that they had been active for at least 60 minutes/day, in the seven days prior to the survey, was three times higher than the proportion of the girls (4.3%).

**Table 1.** Descriptive characteristics of the sample according to sex (n = 102,072)

Variables	n	Total sample % (95% CI)	n	Girls % (95% CI)	n	Boys % (95% CI)
**Age**						
< 14 years	68,871	67.5% (67.2-67.8)	37,968	71.9 (71.5-72.3)	30,903	62.7 (62.3-63.1)
≥ 14 years	33,201	32.5% (32.2-32.8)	14,814	28.1 (27.7-28.5)	18,387	37.3 (36.9-37.7)
Ethnicity						
White	33,775	33.1% (32.8-33.4)	16,779	31.8 (31.4-32.2)	16,996	34.5 (34.1-34.9)
Other	68,189	66.9% (66.6-67.2)	35,956	68.2 (67.8-68.6)	32,233	65.5 (65.1-65.9)
**Bullying**						
No	53,560	52.7% (52.4-53.0)	26,439	50.2 (49.8-50.6)	27,121	55.3 (54.9-55.8)
Yes	48,117	47.3% (47.0-47.6)	26,215	49.8 (49.4-50.2)	21,902	44.7 (44.2-45.1)
**HDI Brazilian regions**						
Low	60,271	59.0% (58.7-59.3)	31,589	59.8 (59.4-60.3)	28,682	58.2 (57.8-58.6)
High	41,801	41.0% (40.7-41.3)	21,193	40.2 (39.7-40.6)	20,608	41.8 (41.4-42.2)
**TV viewing**						
≤ 2 hours	42,635	41.8% (41.6-42.2)	21,543	40.8 (40.5-41.3)	21,092	42.9 (42.5-43.4)
> 2 to ≤ 4 hours	25,819	25.4% (25.1-25.6)	12,829	24.4 (24.0-24.7)	12,990	26.4 (26.1-26.8)
> 4 hours	33,353	32.8% (32.5-33.0)	18,306	34.8 (34.3-35.2)	20,122	30.7 (30.2-31.0)
**PA (days with ≥ 60 minutes)**						
0	34,854	34.3% (34.0-34.6)	23,870	45.5 (45.0-45.9)	10,984	22.4 (22.1-22.8)
1	15886	15.7% (15.4-15.9)	8,651	16.5 (16.2-16.8)	7,235	14.8 (14.5-15.1)
2	13,004	12.8% (12.6-13.0)	6,263	11.9 (11.6-12.2)	6,741	13.8 (13.5-14.1)
3	10,226	10.1% (9.9-10.3)	4,221	8.0 (7.8-8.3)	6,005	12.3 (12.0-12.6)
4	6,281	6.2% (6.0-6.3)	2,376	4.5 (4.3-4.7)	3,905	8.0 (7.7-8.2)
5	7,662	7.5% (7.4-7.7)	3,126	6.0 (5.8-6.2)	4,536	9.3 (9.0-9.5)
6	4,757	4.7% (4.6-4.8)	1,717	3.3 (3.1-3.4)	3,040	6.2 (6.0-6.4)
7	8,836	8.7% (8.5-8.9)	2,295	4.3 (4.2-4.5)	6,541	13.4 (13.1-13.7)
**Insomnia**						
No	89,905	88.5% (88.3-88.7)	44,413	84.4 (84.1-84.7)	45,492	92.9 (92.7-93.1)
Yes	11,699	11.5% (11.3-11.7)	8,230	15.6 (15.3-15.9)	3,469	7.1 (6.9-7.3)

CI = confidence interval; HDI = Human Development Index; PA = physical activity.

In the crude and adjusted associations between daily PA and insomnia, according to sex and HDI of the Brazilian regions (Table 2), it was observed that, compared with their most inactive peers (zero/no active day), girls who accumulated at least 60 minutes/day of physical activity on up to three days/week were less prone to present insomnia, regardless of the region’s HDI. After adjusting the analyses, this pattern of association was maintained only for those who lived in high HDI regions (OR = 0.87; 95% CI = 0.75-0.99). On the other hand, girls who accumulated at least 60 minutes/day of physical activity on seven days presented higher odds of insomnia, even after adjusting the analyses and regardless of the region’s HDI (low HDI: OR = 1.17; 95% CI = 1.01-1.35; high HDI: OR = 1.25; 95% CI = 1.05-1.48). Among the boys, there was a positive association between the number of active days and protection against insomnia, especially for those who lived in high HDI regions.

**Table 2:** Crude and adjusted associations between daily PA (≥ 60 minutes) and insomnia, according to sex and HDI of the Brazilian regions (n = 102,072)

Variables	Girls			
	Low HDI		High HDI	
	Crude OR (95% CI)	Adjusted OR (95% CI)	Crude OR (95% CI)	Adjusted OR (95% CI)
**PA (days with ≥ 60 minutes)**				
0	1	1	1	1
1	0.84 (0.77-0.92)	0.86 (0.78-0.94)	0.73 (0.65-0.82)	0.74 (0.66-0.83)
2	0.87 (0.79-0.97)	0.90 (0.81-0.99)	0.85 (0.75-0.95)	0.87 (0.77-0.98)
3	0.88 (0.78-1.00)	0.90 (0.79-1.02)	0.85 (0.74-0.98)	0.87 (0.75-0.99)
4	1.12 (0.96-1.30)	1.12 (0.97-1.31)	0.97 (0.82-1.15)	0.99 (0.83-1.18)
5	0.93 (0.81-1.07)	0.93 (0.81-1.07)	0.95 (0.81-1.11)	0.96 (0.82-1.12)
6	1.15 (0.97-1.36)	1.13 (0.96-1.34)	1.01 (0.83-1.24)	0.98 (0.80-1.21)
7	1.20 (1.04-1.39)	1.17 (1.01-1.35)	1.28 (1.08-1.52)	1.25 (1.05-1.48)
	**Boys**			
**PA (days with ≥ 60 minutes)**				
0	1	1	1	1
1	0.73 (0.63-0.85)	0.76 (0.66-0.88)	0.69 (0.57-0.83)	0.71 (0.59-0.86)
2	0.70 (0.60-0.82)	0.74 (0.64-0.87)	0.58 (0.47-0.70)	0.61 (0.50-0.74)
3	0.68 (0.57-0.80)	0.72 (0.61-0.85)	0.70 (0.58-0.84)	0.73 (0.60-0.89)
4	0.74 (0.61-0.89)	0.77 (0.63-0.93)	0.76 (0.62-0.94)	0.82 (0.66-1.01)
5	0.77 (0.65-0.92)	0.80 (0.67-0.96)	0.69 (0.56-0.85)	0.73 (0.59-0.91)
6	0.81 (0.66-0.99)	0.83 (0.68-1.02)	0.75 (0.59-0.95)	0.78 (0.61-0.99)
7	0.93 (0.81-1.08)	0.96 (0.83-1.12)	0.79 (0.66-0.94)	0.83 (0.69-0.99)

HDI = Human Development Index; OR = odds ratio; CI = confidence interval; PA = physical activity.

Analysis adjusted for age group, ethnicity, bullying and TV viewing.

## DISCUSSION

The findings of the present study demonstrated that girls who were active for at least 60 minutes/day, on up to two days/week (low HDI) and on up to three days/week (high HDI) presented lower odds of insomnia. However, girls who achieved the daily guidelines for PA on all days presented greater odds of insomnia (regardless of the region’s HDI). For boys, there was a positive association between the number of active days and protection against insomnia, mainly for those who lived in regions with high HDI.

Compared with their most inactive peers (no active day), girls who were active for at least two days/week and boys who were active for at least five days/week presented lower odds of insomnia. These results were similar to previous research conducted among adolescents, which found that higher levels of PA were associated with better sleep quality.^16^^,^^17^^,^^20^ However, we found that this association varied according to the number of active days/week and according to the HDI of the region, which suggested that for this group of schoolchildren the association between PA and sleep did not depend on achievement of the international PA guidelines. This is especially important since previous research had suggested that although the dose-response relationship between PA and mental health outcomes was uncertain, it could occur even at lower doses.^33^^,^^34^^,^^35^ Likewise, a study conducted with a representative sample of European adolescents suggested that even those who did not reach the international PA recommendations, but who showed moderately increased activity, could achieve a meaningful improvement in wellbeing,^36^ which might extend to better sleep quality.

Furthermore, studies that investigated the association between socioeconomic status and sleep quality among adolescents showed that individuals who lived in regions with greater social and economic vulnerability slept less than those who lived in more developed areas.^37^^,^^38^^,^^39^ Thus, the present study has added important information regarding the impact of daily PA on the sleep quality of adolescents, suggesting that social and economic vulnerability may influence not only differences in PA recommendations, but also the association between PA and sleep.

The association between PA and sleep is based on mechanisms such as thermoregulation; increased metabolic rate and energy consumption; changes to heart rate, physical fitness levels and body composition;^15^ and changes to the circadian cycle.^40^ Furthermore, PA is associated with reduced anxiety and depression, increased self-esteem and increased psychological wellbeing, which, in turn, may mediate the relationship between PA and sleep.^40^

Cross-sectional data from a previous survey carried out in the United States showed that only adolescents who were active for ≥ 60 minutes/day, on seven days per week, reported having sufficient amounts of sleep.^18^ On the other hand, we observed that even adolescents who did not reach the weekly PA guidelines,^41^ but who were active on at least two days/week (girls) and five days/week (boys), had better sleep quality, as also pointed out in other research.^15^^,^^19^ In addition, our results reinforce the differences in the association between PA and insomnia according to sex, thus corroborating previous findings,^16^^,^^19^ as well as highlighting the importance of planning tailored interventions that take into account the impact of social and economic vulnerabilities on health outcomes.

One interesting finding was that girls who were active for at least 60 minutes/day, on seven days per week, presented greater odds of insomnia, regardless of the HDI of the region in which they lived. This result is similar to that of previous studies,^20^^,^^36^ in which it was concluded that the subgroup of girls with high PA levels might include adolescents who over-exercise and who are more susceptible to stressors, such as depression symptoms and anxiety,^36^ which are associated with insomnia. Furthermore, an association among girls between over-exercise and sleep disorders has been documented among elite athletes,^42^^,^^43^ such that a high training load was associated with higher prevalence of insomnia, compared with male athletes.^43^

Unfortunately, the PeNSE 2015 data did not allow investigation of the influence of potential mediators, such as social and physical environmental features, like noise and air pollution, traffic, light exposure and safety, which can mediate the relationship between PA and sleep quality.^27^^,^^28^ Furthermore, the association among girls between over-exercise and insomnia occurred regardless of the region’s HDI, and persisted even after controlling for possible confounding variables such as age, ethnicity, bullying and TV viewing, thus suggesting that other mediators that were not evaluated through PeNSE 2015 may be involved. Future research should investigate other possible mediators between excessive PA and insomnia, especially among girls.

Despite the relevance of the results highlighted here, the present study had some limitations. First, the data analyzed were based on questionnaires, which are more subject to recall bias. Nevertheless, questionnaires are viable low-cost instruments and have become well established in epidemiological research worldwide.^44^ Second, subjective sleep assessment did not allow analysis of other sleep outcomes, such as bedtime, sleep onset latency, waking time after sleep onset and total sleep time, or their possible associations with PA.^15^ Third, the dichotomization of the study variables may have attenuated the power to detect associations.^45^

However, the study also had some strengths. First, this was one of the few studies to analyze the association between daily PA and insomnia among adolescents who were not living in developed countries, and it also took social and economic vulnerability into consideration. Second, given that this survey was based on a sample with national representation, the results can be generalized for the Brazilian population of school-age adolescents.^46^

## CONCLUSION

Even amounts of physical activity that were lower than the weekly guidelines were associated with better sleep quality for Brazilian adolescents, especially girls, and even for those who were living in regions with greater social and economic vulnerability. Thus, the present study has important implications for policy makers. It highlights the need to consider regional inequalities and it shows that physical activity-based interventions can be targeted as a low-cost, non-pharmacological and widely viable alternative that may foster better sleep quality among adolescents. Further research should encourage use of prospective data to better understand the relationship between daily physical activity and sleep quality, taking into account the influence of social and economic vulnerability.
